# Supplementary material for: Human substance P receptor binding mode of the antagonist drug aprepitant by NMR and crystallography
Source: Nat Commun. 2019 Feb 7;10:638. doi: 10.1038/s41467-019-08568-5 (PMC6367319; doi:10.1038/s41467-019-08568-5)
Supplement: Supplementary file 1 — Supplementary Information [file 41467_2019_8568_MOESM1_ESM.pdf]

Supplementary Information

Human substance P receptor binding mode of the antagonist drug aprepitant by NMR and crystallography

S. Chen, M. Lu, D. Liu et al.

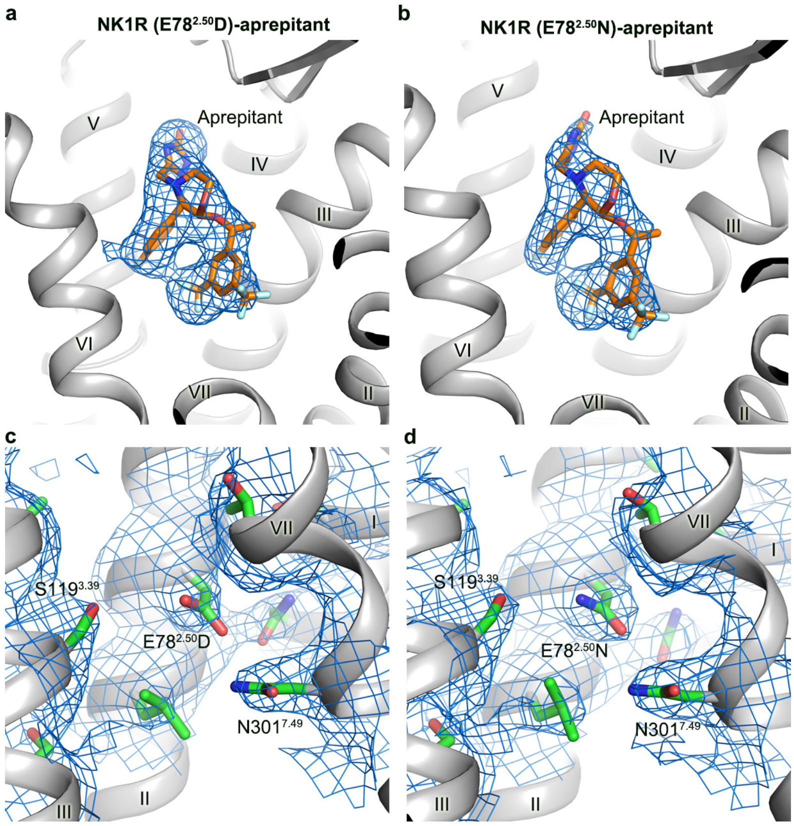

Supplementary Figure 1 | Electron densities of ligand and residues around position 2.50 in NK1R structures. a and **b**, Electron densities of aprepitant in the NK1R (E78$^{2.50}$D)-aprepitant structure (a) and NK1R (E78$^{2.50}$N)-aprepitant structure (b). The NK1R structure is shown in grey. The ligand is displayed as sticks with orange carbons. Electron densities are contoured at 3.0 σ from a |*Fo*| - |*Fc*| omit map and coloured blue. **c** and **d**, Electron densities of the residues around the position 2.50 in the NK1R (E78$^{2.50}$D)-aprepitant structure (c) and NK1R (E78$^{2.50}$N)-aprepitant structure (d). The NK1R structure is shown in grey. The residues around the position 2.50 are displayed as green sticks. Electron densities are contoured at 1.0 σ from a |2*Fo*| - |*Fc*| map and coloured blue.

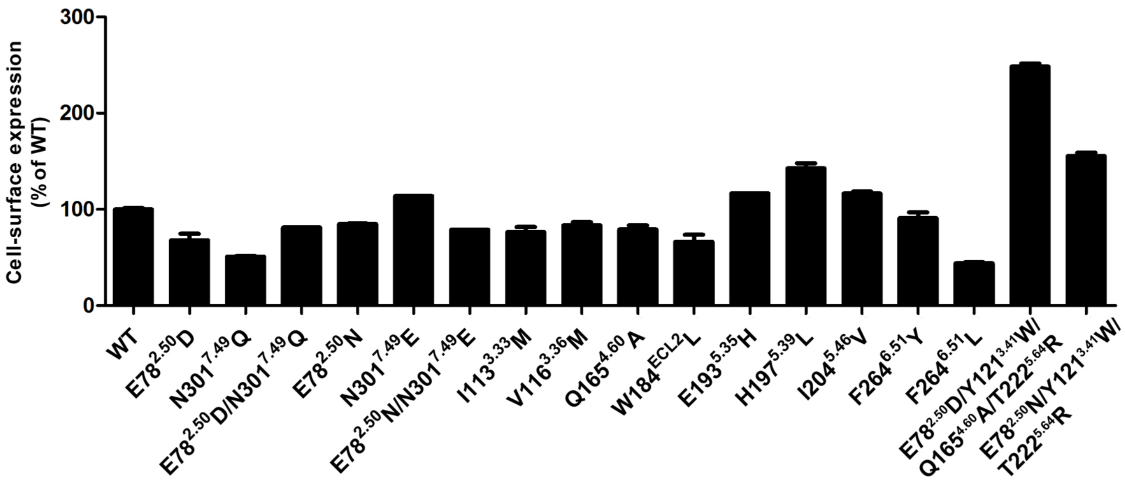

Supplementary Figure 2 | Cell-surface expression of wild-type (WT) and mutant NK1 receptors in HEK293 cells. Data are shown as mean ± s.e.m. from three independent experiments using independently transfected cells and performed in triplicate. Source data are provided as a Source Data file.

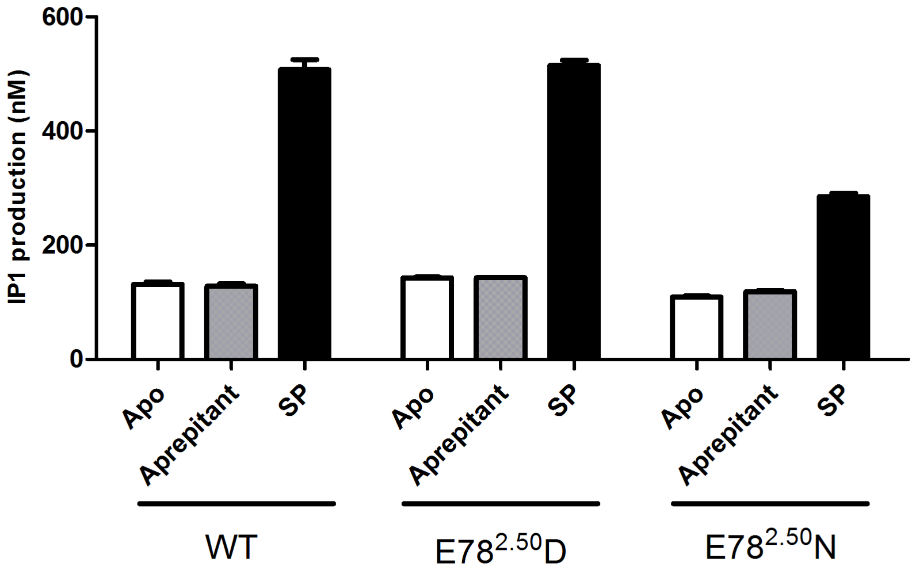

Supplementary Figure 3 | IP1 accumulation of wild-type NK1R (WT) and the E78$^{2.50}$D and E78$^{2.50}$N mutants in apo state and in the presence of aprepitant (at 1 μM concentration) or SP (at 1 μM concentration). Data shown are mean ± s.e.m. from at least three independent experiments performed in technical triplicate. For E78$^{2.50}$N-apo and E78$^{2.50}$N-aprepitant, three independent experiments were performed. For the other samples, four independent experiments were performed. Source data are provided as a Source Data file.

a

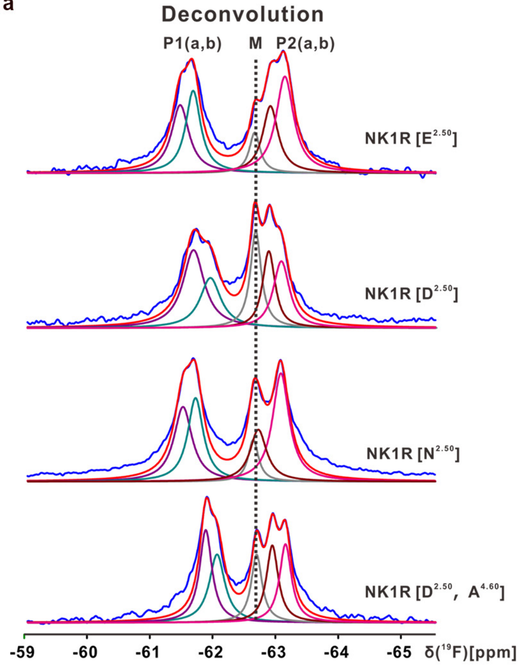

b

Integral signal intensities (%)

	P1a	P1b	M	P2a	P2b
NK1R [$E^{2.50}$]	21.5	22	8	18.5	30
NK1R [$D^{2.50}$]	29	18	18.5	16	18.5
NK1R [$N^{2.50}$]	23	23	8	16	30
NK1R [$D^{2.50}$, $A^{4.60}$]	22	24	15.5	19	19.5

Supplementary Figure 4 | Lorentzian deconvolution of the 1D ^{19}F-NMR spectra. a, NMR spectra of aprepitant bound to four different NK1R mutants, as indicated. The blue line represents the experimental ^{19}F-NMR signal. The components obtained by deconvolution are displayed with the following colour code: P1a, purple; P1b, green; M, grey; P2a, brown; P2b, pink; sum of the five deconvoluted peaks, red. **b**, Relative intensities of the component peaks.

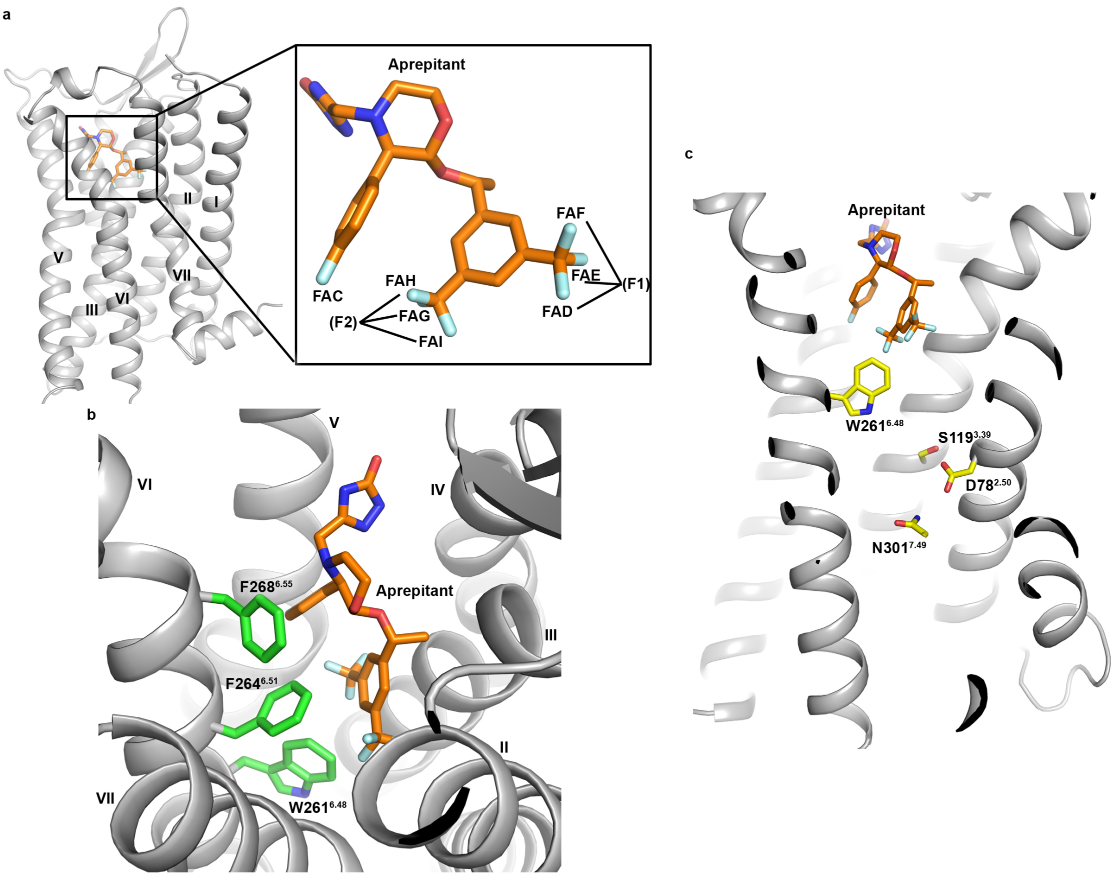

Supplementary Figure 5 | Origin of ring current shifts for the trifluoromethyl group bound to aprepitant in NK1R and interaction network of aprepitant to NK1R. a, Notation of the trifluoromethyl groups in aprepitant. **b,** Location of aprepitant relative to the NK1R residues W261$^{6.48}$, F264$^{6.51}$, and F268$^{6.55}$ that induce major ring current shifts of the trifluoromethyl groups. **c,** The corresponding positions of aprepitant to key residues of receptor activation.

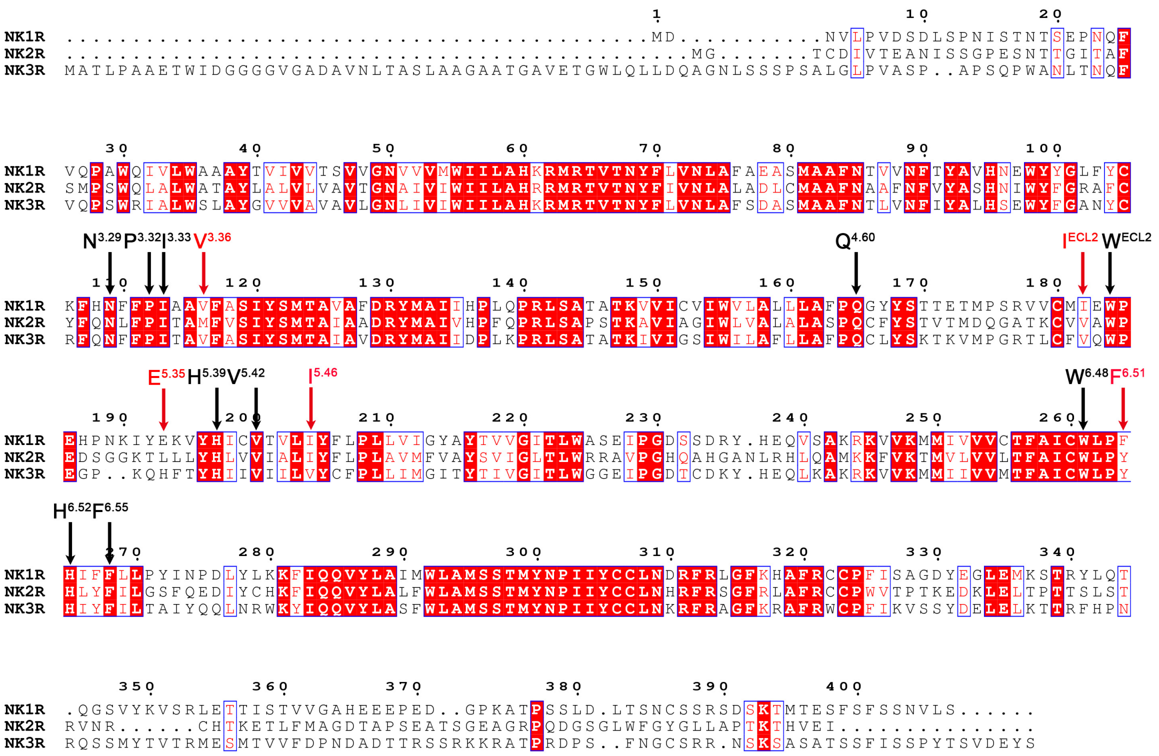

Supplementary Figure 6 | Sequence alignment of the human neurokinin receptors. Colours represent the degree of similarity of residues: red background, identical; red text, similar. Key residues in the aprepitant binding pocket, which are conserved or variable among receptors, are indicated by black and red arrows, respectively. The alignment was generated by uniprot (http://www.uniprot.org/align/) and the graphic was prepared on the ESPript 3.0 server (http://espript.ibcp.fr/ESPript/cgi-bin/ESPript.cgi).

Supplementary Table 1 | Data collection and structure refinement statistics.

	NK1R ($E78^{2.50}$D)-aprepitant	NK1R ($E78^{2.50}$N)-aprepitant
**Data collection**[a]		
Space group	$P4_22_12$	$P4_22_12$
Cell dimensions		
a, b, c (Å)	103.1, 103.1, 158.2	102.5, 102.5, 157.0
α, β, γ (°)	90.0, 90.0, 90.0	90.0, 90.0, 90.0
Resolution (Å)	50.0-3.20 (3.37-3.20)[b]	50.0-2.70 (2.83-2.70)
R_{merge}	0.15 (0.77)	0.13 (0.99)
$I/\sigma(I)$	9.8 (2.1)	8.3 (1.1)
$CC_{1/2}$	0.99 (0.77)	0.99 (0.50)
Completeness (%)	92 (60)	95 (60)
Redundancy	12.1 (9.8)	9.2 (3.8)
**Refinement**		
Resolution (Å)	50.0-3.20	50.0-2.70
No. reflections	13,746	23,622
R_{work} / R_{free}	0.214 / 0.254	0.233 / 0.261
No. atoms		
Protein	3,115	3,147
Ligand	37	37
Others	19	0
B factors (Å^2)		
NK1R	125.6	117.4
mT4L	116.6	108.2
Ligand	107.7	99.5
R.m.s. deviations		
Bond lengths (Å)	0.010	0.009
Bond angles (°)	1.04	1.01

[a]Diffraction data from 47 NK1R ($E78^{2.50}$D)-aprepitant crystals and 21 NK1R ($E78^{2.50}$N)-aprepitant crystals were used to solve the structures.

[b]Values in parentheses are for highest-resolution shell.

Supplementary Table 2 | IP1 accumulation assays of wild-type (WT) and mutant NK1 receptors for the agonist SP and inhibition by aprepitant.

	SP[a]			SP/aprepitant[b]			
WT/mutants	EC_{50} (nM)	pEC_{50} ± s.e.m.[c]	n[d]	EC_{50} (nM)	pEC_{50} ± s.e.m.	n	Ratio[e]
WT	2.4	8.63 ± 0.06	4	117	6.93 ± 0.23	4	49
$E78^{2.50}D$[f]	2.7	8.57 ± 0.01	3	64	7.19 ± 0.02	3	24
$N301^{7.49}Q$	2.6	8.58 ± 0.02	3	71	7.15 ± 0.04	3	27
$E78^{2.50}D/N301^{7.49}Q$	2.7	8.57 ± 0.02	3	68	7.17 ± 0.05	3	25
$E78^{2.50}N$	2.5	8.60 ± 0.03	3	254	6.60 ±0.27	3	102
$N301^{7.49}E$	4.0	8.39 ± 0.08	3	119	6.93 ±0.93	3	30
$E78^{2.50}N/N301^{7.49}E$	2.5	8.61 ± 0.05	3	79	7.10 ± 0.03	3	32
$I113^{3.33}M$	3.1	8.52 ± 0.05	3	41	7.39 ±0.09	3	13
$V116^{3.36}M$	2.8	8.55 ± 0.05	3	38	7.42 ±0.10	3	14
$Q165^{4.60}A$	2.2	8.65 ± 0.03	3	27	7.56 ±0.11	3	12
$W184^{ECL2}L$	2.4	8.62 ± 0.07	3	47	7.32 ±0.11	3	20
$E193^{5.35}H$	2.1	8.68 ± 0.05	3	27	7.57 ±0.20	3	13
$H197^{5.39}L$	3.2	8.50 ± 0.02	3	101	7.00 ±0.19	3	32
$I204^{5.46}V$	2.7	8.57 ± 0.03	3	17	7.78 ±0.23	3	6.2
$F264^{6.51}Y$	2.9	8.53 ± 0.02	3	18	7.74 ±0.11	3	6.2
$F264^{6.51}L$	3.0	8.52 ± 0.06	3	46	7.34 ±0.14	3	15
$E78^{2.50}D/Y121^{3.41}W/Q165^{4.60}A/T222^{5.64}R$	3.0	8.52 ± 0.06	3	219	6.66 ± 0.15	3	73
$E78^{2.50}N/Y121^{3.41}W/T222^{5.64}R$	4.3	8.37 ± 0.02	3	163	6.79 ± 0.75	3	38

[a]EC_{50} values were determined after 0.5 h stimulation by increasing concentrations of SP.

[b]EC_{50} values were determined after 0.5 h stimulation by increasing concentrations of SP together with 100 nM aprepitant.

[c]Data represent mean ± s.e.m.

[d]Sample size; the number of independent experiments performed in technical triplicate or duplicate.

[e]The EC_{50} ratio represents the shift between the SP and SP + aprepitant curve (EC_{50}(SP + aprepitant)/EC_{50}(SP)) and characterizes the antagonistic effect on the WT receptor or receptor mutants. By comparison of EC_{50} ratios between WT and mutant receptors, influences of all tested residues on antagonist activity were determined. A higher ratio indicates higher antagonist activity. A reduced EC_{50} ratio of mutant compared to the wild-type receptor was interpreted as important for the respective antagonist.

[f]All mutations were introduced to the WT NK1R.

Supplementary Table 3 | cAMP assays of wild-type (WT) and mutant NK1 receptors for the agonist SP.

WT/mutants	EC_{50} (nM)	pEC_{50} ± s.e.m.[a]	n[b]
WT	4.1	8.38 ± 0.04	4
E78$^{2.50}$D[b]	8.0	8.10 ± 0.07	3
N301$^{7.49}$Q	2.3	8.64 ± 0.06	4
E78$^{2.50}$D/N301$^{7.49}$Q	3.1	8.52 ± 0.04	3
E78$^{2.50}$N	18	7.76 ± 0.08	3
N301$^{7.49}$E	3.0	8.53 ± 0.13	3
E78$^{2.50}$N/N301$^{7.49}$E	2.3	8.65 ± 0.05	3

[a]Data represent mean ± s.e.m.

[b]Sample size; the number of independent experiments performed in technical triplicate.

[c]All mutations were introduced to the WT NK1R.

Supplementary Table 4 | ^{19}F ring current shifts for aprepitant in NK1R complexes.

Mutant	Δδ(ppm)[a]	Pseudo atom[b]	δ_R(ppm)[c]	Contributing residues (δ_R in ppm)[d]
$E^{2.50}D$	0.73 (P1)	F1	0.12	W261 (0.16), F264 (-0.11)
	-0.34 (P2)	F2	-0.18	W261 (-0.14), F268 (-0.10)
$E^{2.50}N$	1.04 (P1)	F1	0.28	W261 (0.19)
	-0.38 (P2)	F2	-0.29	W261 (-0.24), F268 (-0.06)

[a]Observed chemical shift difference between receptor (P1, P2 in Fig. 4) and micelle-bound (M) peaks. Average values of the two polymorphism peaks are used (see Supplementary Figure 4).

[b]Ring current shifts for the trifluoromethyl groups were calculated for the pseudo-atom positions of F1, and F2 representing the three ^{19}F atoms. The Johnson–Bovey algorithm[1] in the program MOLMOL[2] was used.

[c]Calculated ring current shifts.

[d]NK1R residues with major contributions to δ_R.

Supplementary Table 5 | DNA sequence of the codon-optimized NK1R gene and primer sequences.

NK1R (E78$^{2.50}$D) gene sequence
GATAACGTCCTCCCGGTGGACTCAGACCTCTCCCCAAACATCTCCACTAACACCTCGG
AACCCAATCAGTTCGTGCAACCAGCCTGGCAAATTGTCCTTTGGGCAGCTGCCTACAC
GGTCATTGTGGTGACCTCTGTGGTGGGCAACGTGGTAGTGATGTGGATCATCTTAGCC
CACAAAAGAATGAGGACAGTGACGAACTATTTTCTGGTGAACCTGGCCTTCGCGGAT
GCCTCCATGGCTGCATTCAATACAGTGGTGAACTTCACCTATGCTGTCCACAACGAAT
GGTACTACGGCCTGTTCTACTGCAAGTTCCACAACTTCTTTCCCATCGCCGCTGTCTTC
GCCAGTATCTGGTCCATGACGGCTGTGGCCTTTGATAGGTACATGGCCATCATACATC
CCCTCCAGCCCCGGCTGTCAGCCACAGCCACCAAAGTGGTCATCTGTGTCATCTGGGT
CCTGGCTCTCCTGCTGGCCTTCCCCGCGGGCTACTACTCAACCACAGAGACCATGCCC
AGCAGAGTCGTGTGCATGATCGAATGGCCAGAGCATCCGAACAAGATTTATGAGAAA
GTGTACCACATCTGTGTGACTGTGCTGATCTACTTCCTCCCCCTGCTGGTGATTGGCTA
TGCATACACCGTAGTGGGAATCAGACTATGGGCTAGCAACATCTTCGAGATGCTGCGT
ATCGATGAGGGAGGCGGCAGTGGCGGCGACGAGGCCGAAAAGTTGTTCAACCAGGA
TGTGGACGCCGCCGTACGTGGAATTTTGCGTAACGCGAAGCTGAAGCCTGTCTATGAT
AGCCTGGATGCTGTCAGACGTGCAGCCCTGATTAACATGGTGTTCCAAATGGGCGAG
ACGGGAGTCGCAGGCTTCACCAATTCGCTCAGGATGCTCCAGCAAAAGCGTTGGGAT
GAAGCAGCGGTCAACTTGGCAAAGTCCAGATGGTACAACCAGACCCCTAACAGAGCC
AAACGTGTGATCACCACCTTCAGGACCGGTACATGGGACGCTTACCACGAGCAAGTC
TCTGCCAAGCGCAAGGTGGTCAAAATGATGATTGTCGTGGTGTGCACCTTCGCCATCT
GCTGGCTGCCCTTCCACATCTTCTTCCTCCTGCCCTACATCAACCCAGATCTCTACCTG
AAGAAGTTTATCCAGCAGGTCTACCTGGCCATCATGTGGCTGGCCATGAGCTCCACCA
TGTACAACCCCATCATCTACTGCTGCCTCAATGACAGGTTCCGTCTGGGCTTCAAGCA
TGCCTTCCGGTGCTGCCCCTTCATCAGCGCCGGCGACTATGAGGGGCTGGAA
NK1R (E78$^{2.50}$N) gene sequence
GATAACGTCCTCCCGGTGGACTCAGACCTCTCCCCAAACATCTCCACTAACACCTCGG
AACCCAATCAGTTCGTGCAACCAGCCTGGCAAATTGTCCTTTGGGCAGCTGCCTACAC
GGTCATTGTGGTGACCTCTGTGGTGGGCAACGTGGTAGTGATGTGGATCATCTTAGCC
CACAAAAGAATGAGGACAGTGACGAACTATTTTCTGGTGAACCTGGCCTTCGCGAAC
GCCTCCATGGCTGCATTCAATACAGTGGTGAACTTCACCTATGCTGTCCACAACGAAT
GGTACTACGGCCTGTTCTACTGCAAGTTCCACAACTTCTTTCCCATCGCCGCTGTCTTC
GCCAGTATCTGGTCCATGACGGCTGTGGCCTTTGATAGGTACATGGCCATCATACATC
CCCTCCAGCCCCGGCTGTCAGCCACAGCCACCAAAGTGGTCATCTGTGTCATCTGGGT
CCTGGCTCTCCTGCTGGCCTTCCCCCAGGGCTACTACTCAACCACAGAGACCATGCCC
AGCAGAGTCGTGTGCATGATCGAATGGCCAGAGCATCCGAACAAGATTTATGAGAAA
GTGTACCACATCTGTGTGACTGTGCTGATCTACTTCCTCCCCCTGCTGGTGATTGGCTA
TGCATACACCGTAGTGGGAATCAGACTATGGGCTAGCAACATCTTCGAGATGCTGCGT
ATCGATGAGGGAGGCGGCAGTGGCGGCGACGAGGCCGAAAAGTTGTTCAACCAGGA
TGTGGACGCCGCCGTACGTGGAATTTTGCGTAACGCGAAGCTGAAGCCTGTCTATGAT

AGCCTGGATGCTGTCAGACGTGCAGCCCTGATTAACATGGTGTTCCAAATGGGCGAG ACGGGAGTCGCAGGCTTCACCAATTCGCTCAGGATGCTCCAGCAAAAGCGTTGGGAT GAAGCAGCGGTCAACTTGGCAAAGTCCAGATGGTACAACCAGACCCCTAACAGAGCC AAACGTGTGATCACCACCTTCAGGACCGGTACATGGACGCTTACCACGAGCAAGTC TCTGCCAAGCGCAAGGTGGTCAAAATGATGATTGTCGTGGTGTGCACCTTCGCCATCT GCTGGCTGCCCTTCCACATCTTCTTCCTCCTGCCCTACATCAACCCAGATCTCTACCTG AAGAAGTTTATCCAGCAGGTCTACCTGGCCATCATGTGGCTGGCCATGAGCTCCACCA TGTACAACCCCATCATCTACTGCTGCCTCAATGACAGGTTCCGTCTGGGCTTCAAGCA TGCCTTCCGGTGCTGCCCCTTCATCAGCGCCGGCGACTATGAGGGGCTGGAA	
Primer sequences	
pFastbac-N-F	ATTGGCGCGCCGGATAACGTCCTCCCGGTG
pFastbac-C-R	AAAGGCCGGCCGGAGAGCACATTGGAGGAGAA
pFastbac-C-truncation-R	AAAGGCCGGCCTTCCAGCCCCTCATA
pcDNA3.1-N-F	GGAAAGCTTGCCACCATGGACTACAAGGACGATGATGAC GATAACGTCCTCCCGGTGGA
pcDNA3.1-C-R	TCCGAATTCGGAGAGCACATTGGAGGAGAA
pcDNA3.1-C-truncation-R	TCCGAATTCTTCCAGCCCCTCATAGTCGCC
ICL3-mT4L-N-F	ATCACTCTATGGGCTAGCAACATCTTCGAGATGCTG
ICL3-mT4L-N-R	CAGCATCTCGAAGATGTTGCTAGCCCATAGAGTGAT
ICL3-mT4L-C-F	GGTACATGGGACGCTTACCACGAGCAAGTCTCTGCC
ICL3-mT4L-C-R	GGCAGAGACTTGCTCGTGGTAAGCGTCCCATGTACC
$E78^{2.50}$D-F	GTGAACCTGGCCTTCGCGGACGCCTCCATGGCTGCATTC
$E78^{2.50}$D-R	GAATGCAGCCATGGAGGCGTCCGCGAAGGCCAGGTTCAC
$E78^{2.50}$N-F	GTGAACCTGGCCTTCGCGAACGCCTCCATGGCTGCATTC
$E78^{2.50}$N-R	GAATGCAGCCATGGAGGCGTTCGCGAAGGCCAGGTTCAC
$I113^{3.33}$M-F	CACAACTTCTTTCCCATGGCCGCTGTCTTCGCC
$I113^{3.33}$M-R	GGCGAAGACAGCGGCCATGGGAAAGAAGTTGTG
$V116^{3.36}$M-F	TTTCCCATCGCCGCTATGTTCGCCAGTATCTAC
$V116^{3.36}$M-R	GTAGATACTGGCGAACATAGCGGCGATGGGAAA
$Y121^{3.41}$W-F	GTCTTCGCCAGTATCTGGTCCATGACGGCTGTG
$Y121^{3.41}$W-R	CACAGCCGTCATGGACCAGATACTGGCGAAGAC
$Q165^{4.60}$A-F	CTGCTGGCCTTCCCCGCCGGCTACTACTCAACC
$Q165^{4.60}$A-R	GGTTGAGTAGTAGCCGGCGGGGAAGGCCAGCAG
$W184^{ECL2}$L-F	GTGTGCATGATCGAACTGCCAGAGCATCCGAAC
$W184^{ECL2}$L-R	GTTCGGATGCTCTGGCAGTTCGATCATGCACAC
$E193^{5.35}$H-F	CCGAACAAGATTTATCACAAAGTGTACCACATC
$E193^{5.35}$H-R	GATGTGGTACACTTTGTGATAAATCTTGTTCGG
$H197^{5.39}$L-F	TATGAGAAAGTGTACCTGATCTGTGTGACTGTG
$H197^{5.39}$L-R	CACAGTCACACAGATCAGGTACACTTTCTCATA
$I204^{5.46}$V-F	TGTGTGACTGTGCTGGTGTACTTCCTCCCCCTG
$I204^{5.46}$V-R	CAGGGGGAGGAAGTACACCAGCACAGTCACACA
$T222^{5.64}$R-F	ACCGTAGTGGGAATCCGTCTATGGGCTAGC

T222$^{5.64}$R-R	GCTAGCCCATAGACGGATTCCCACTACGGT
F264$^{6.51}$Y-F	ATCTGCTGGCTGCCCTACCACATCTTCTTCCTC
F264$^{6.51}$Y-R	GAGGAAGAAGATGTGGTAGGGCAGCCAGCAGAT
F264$^{6.51}$L-F	ATCTGCTGGCTGCCCCTCCACATCTTCTTCCTC
F264$^{6.51}$L-R	GAGGAAGAAGATGTGGAGGGGCAGCCAGCAGAT
N301$^{7.49}$Q-F	ATGAGCTCCACCATGTACCAGCCCATCATCTACTGCTGC
N301$^{7.49}$Q-R	GCAGCAGTAGATGATGGGCTGGTACATGGTGGAGCTCAT
N301$^{7.49}$E-F	ATGAGCTCCACCATGTACGAACCCATCATCTACTGCTGC
N301$^{7.49}$E-R	GCAGCAGTAGATGATGGGTTCGTACATGGTGGAGCTCAT

Supplementary References

1. Johnson CE, Bovey FA. Calculation of Nuclear Magnetic Resonance Spectra of Aromatic Hydrocarbons. *J Chem Phys* **29**, 1012-1014 (1958).
2. Koradi R, Billeter M, Wuthrich K. MOLMOL: a program for display and analysis of macromolecular structures. *Journal of molecular graphics* **14**, 51-55, 29-32 (1996).
