# Supplementary material for: Human substance P receptor binding mode of the antagonist drug aprepitant by NMR and crystallography
Source: Nat Commun. 2019 Feb 7;10:638. doi: 10.1038/s41467-019-08568-5 (PMC6367319; doi:10.1038/s41467-019-08568-5)
Supplement: Supplementary file 3 — Reporting Summary [file 41467_2019_8568_MOESM3_ESM.pdf]

nature research

Corresponding author(s): Qiang Zhao, Kurt Wüthrich and Beili Wu

Last updated by author(s): Dec 29, 2018

Reporting Summary

Nature Research wishes to improve the reproducibility of the work that we publish. This form provides structure for consistency and transparency in reporting. For further information on Nature Research policies, see Authors & Referees and the Editorial Policy Checklist.

Statistics

For all statistical analyses, confirm that the following items are present in the figure legend, table legend, main text, or Methods section.

n/a	Confirmed	
☐	☒	The exact sample size (*n*) for each experimental group/condition, given as a discrete number and unit of measurement
☐	☒	A statement on whether measurements were taken from distinct samples or whether the same sample was measured repeatedly
☒	☐	The statistical test(s) used AND whether they are one- or two-sided<br>*Only common tests should be described solely by name; describe more complex techniques in the Methods section.*
☒	☐	A description of all covariates tested
☒	☐	A description of any assumptions or corrections, such as tests of normality and adjustment for multiple comparisons
☐	☒	A full description of the statistical parameters including central tendency (e.g. means) or other basic estimates (e.g. regression coefficient) AND variation (e.g. standard deviation) or associated estimates of uncertainty (e.g. confidence intervals)
☒	☐	For null hypothesis testing, the test statistic (e.g. *F*, *t*, *r*) with confidence intervals, effect sizes, degrees of freedom and *P* value noted<br>*Give P values as exact values whenever suitable.*
☒	☐	For Bayesian analysis, information on the choice of priors and Markov chain Monte Carlo settings
☒	☐	For hierarchical and complex designs, identification of the appropriate level for tests and full reporting of outcomes
☒	☐	Estimates of effect sizes (e.g. Cohen's *d*, Pearson's *r*), indicating how they were calculated

Our web collection on statistics for biologists contains articles on many of the points above.

Software and code

Policy information about availability of computer code

Data collection	No software was used.
Data analysis	Data of cAMP and inositol phosphate (IP) accumulation assays were analyzed using Prism 7 (GraphPad, San Diego, USA).

For manuscripts utilizing custom algorithms or software that are central to the research but not yet described in published literature, software must be made available to editors/reviewers. We strongly encourage code deposition in a community repository (e.g. GitHub). See the Nature Research guidelines for submitting code & software for further information.

Data

Policy information about availability of data

All manuscripts must include a data availability statement. This statement should provide the following information, where applicable:

- Accession codes, unique identifiers, or web links for publicly available datasets
- A list of figures that have associated raw data
- A description of any restrictions on data availability

Atomic coordinates and structure factors of NK1R (E2.50N)-aprepitant and NK1R (E2.50D)-aprepitant complex structures have been deposited in the Protein Data Bank with accession codes 6J20 and 6J21. The source data underlying Fig 3b, c and Supplementary Figs 2 and 3 are provided as a Source Data file. Other data are available from the corresponding authors upon reasonable request.

Field-specific reporting

Please select the one below that is the best fit for your research. If you are not sure, read the appropriate sections before making your selection.

☒ Life sciences ☐ Behavioural & social sciences ☐ Ecological, evolutionary & environmental sciences

For a reference copy of the document with all sections, see nature.com/documents/nr-reporting-summary-flat.pdf

Life sciences study design

All studies must disclose on these points even when the disclosure is negative.

Sample size	Due to radiation damage, X-ray diffraction data collection of the protein crystals was limited to 5-10 degree per crystal. To collect a complete data set for structure determination, diffraction data from multiple crystals were integrated and scaled using XDS. By calculating completeness of the data set, diffraction data from 47 NK1R (E782.50D)-aprepitant crystals and 21 NK1R (E782.50N)-aprepitant crystals were used to ensure the completeness was close to 100%. For the cAMP and IP accumulation assays, at least 3 independent experiments were performed in technical triplicate to ensure each data point was repeatable.
Data exclusions	No data were excluded from the analyses.
Replication	All attempts at replication were successful.
Randomization	Randomization is not relevant to this study, as protein and crystal samples are not required to be allocated into experimental groups in protein structural studies, and no animals or human research participants are involved in this study.
Blinding	Blinding is not relevant to this study, as protein and crystal samples are not required to be allocated into experimental groups in protein structural studies, and no animals or human research participants are involved in this study.

Reporting for specific materials, systems and methods

We require information from authors about some types of materials, experimental systems and methods used in many studies. Here, indicate whether each material, system or method listed is relevant to your study. If you are not sure if a list item applies to your research, read the appropriate section before selecting a response.

Materials & experimental systems

n/a	Involved in the study	
☐	☒	Antibodies
☐	☒	Eukaryotic cell lines
☒	☐	Palaeontology
☒	☐	Animals and other organisms
☒	☐	Human research participants
☒	☐	Clinical data

Methods

n/a	Involved in the study	
☒	☐	ChIP-seq
☒	☐	Flow cytometry
☒	☐	MRI-based neuroimaging

Antibodies

Antibodies used	Monoclonal ANTI-FLAG M2-FITC antibody: Sigma, Cat#F4049; lot number: SLBV7803;<br>Cryptate-labelled anti-IP1 monoclonal antibody: CisBio Bioassays, Cat#62IPAPEC; lot number: 12A.
Validation	Monoclonal ANTI-FLAG M2-FITC antibody: validation statements are available on the manufacturer's website (https://www.sigmaaldrich.com/catalog/product/sigma/f4049?lang=en&region=US);<br>Cryptate-labelled anti-IP1 monoclonal antibody: validation statements are available in the papers titled "IP-3/IP-1 Assays - Assay Guidance Manual" (Pubmed ID 22553873) and "Development of an Improved IP1 Assay for the Characterization of 5-HT2C Receptor Ligands" (Pubmed ID: 19922239).

Eukaryotic cell lines

Policy information about cell lines

Cell line source(s)	Sf9 cells were obtained from Invitrogen, and HEK293 cells were obtained from ATCC.
Authentication	n/a
Mycoplasma contamination	The cell lines are negative for mycoplasma contamination.

Commonly misidentified lines<br>(See ICLAC register)	No commonly misidentified cell lines were used.
